# Fragile site instability: measuring more than breaks

**DOI:** 10.18632/oncoscience.513

**Published:** 2020-06-08

**Authors:** Irina Waisertreiger, Jacqueline Barlow

**Affiliations:** ^1^Department of Microbiology and Molecular Genetics, University of California, Davis, CA, USA; ^2^Department of Microbiology and Molecular Genetics & Genome Center, University of California, Davis, CA, USA

**Keywords:** fluorescent hybridization in situ, genome instability, DNA damage replication stress, homologous recombination repair, sister chromatid exchanges, common fragile site, early replicating fragile site

## Abstract

Genome instability is not only a hallmark of cancer, it is necessary for its initiation and evolution, and naturally accumulates as cells age. Replication stress is a potent source of genome instability found in many tumor types [1]. Chromosomal fragile sites are genomic loci highly prone to DNA damage specifically from replication stress and are frequently mutated in cancer [2-4]2-4]. While tracking the origin of individual mutations has proved challenging, measuring DNA damage and repair at endogenous sites can offer key insights into understanding the etiology of cancer.

In the past 15 years, the causal link between replication stress, oncogene activation, and tumor initiation and evolution has become increasingly clear [1, 5-9]. Replication-associated damage accumulates at early stages of tumorigenesis and may promote further transformation. Studying the causes and consequences of fragile site instability can offer a window into the earliest stages of carcinogenesis [10-13]. In particular, fragile site studies will help us understand the molecular underpinnings influencing the frequency of DNA breakage, successful repair processes suppressing genome instability, and unsuccessful repair leading to mutations and chromosome rearrangements. Of these, measuring successful repair is the most challenging as it leaves little evidence behind.

## Existing methods measuring unsuccessful and successful DNA repair

Here we focus on the repair of DNA double strand breaks (DSBs), a potent source of DNA damage and a critical intermediate in the formation of chromosome rearrangements [14, 15]. DSBs are a common intermediate of replication stress and can be repaired either by non-homologous end-joining (NHEJ) or homologous recombination (HR). Multiple methods exist to measure “unsuccessful” repair—unrepaired DSBs can be directly identified by ligation-mediated PCR or deep sequencing techniques such as LAM-HTGTS, BLESS, BREAK-Seq, and END-Seq [16-20]. Alternatively, mutagenic repair products can be identified by reporter assays, PCR-based insertion-deletion assays, or deep sequencing for unfaithful end joining [19, 21-24]. DNA sequencing can give information on mutagenic repair – however it misses long-range rearrangements. Of note, fluorescent reporter assays are powerful tools that utilize common mistakes in DNA repair to assess the efficiency of specific repair pathways—specific deletion or recombination events restore expression of a fluorescent or selectable marker [22, 23, 25]. Importantly, reporter assays provide critical information on repair frequency—information lost with most PCR- and sequencing-based approaches.

Measuring DNA damage and mutagenesis is straightforward, however “successful” repair is difficult to assess. By their nature, truly faithful NHEJ and HR are invisible leaving no telltale mutations to indicate repair process type; therefore, how do we measure when cells get the job done right? Traditional methods of monitoring DNA breakage and repair by genomic DNA blotting remains the gold-standard as it measures broken DNA and repair product formation [24]. The largest challenge in measuring endogenous damage is knowing where the damage occurs—all current methods require positional information of where the damage takes place to develop PCR primers or probes to assess breakage and repair. To utilize these methods effectively, DNA damage must be confined to a discrete region that can be assessed by standard gel electrophoresis (normally 20 kb or less), or easily amplified by PCR. However endogenous fragile sites induced by replication stress cover very large regions, some have breaks spanning regions larger than 1 Mb [3, 26-29]. Measuring successful fragile site repair by PCR, genomic blots, or DNA sequencing has been unsuccessful due to their size. Finally, only metaphase spread analysis reveals the physical structure of chromosomes harboring complex chromosome rearrangements.

**Figure 1:**
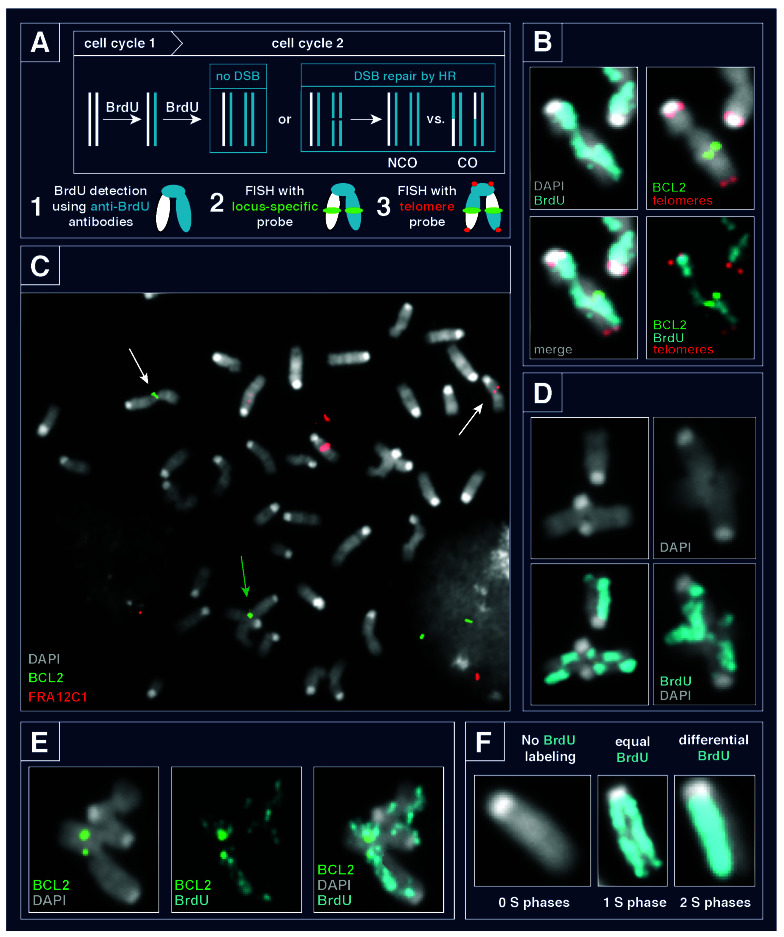
SCE-FISH and its advantages. **A.** SCE-FISH assay scheme. SCE is an event where the two strands of DNA exchange after repair of a DSB, resulting in a crossover event. SCEs can be visualized by differentially labeling the two sister chromatids with the nucleotide analog BrdU. Combining single locus FISH with BrdU staining to measure SCE events allows the measurement of successful DSB repair at a specific locus on a single cell level. Telomere probe visualizes chromosome ends and facilitates cytogenetic analysis of DNA damage. *FISH probes are shown in green, telomere-specific probe is in red, and BrdU shown in cyan*. **B.** SCE-FISH validation showing a SCE at the ERFS locus BCL2 and break colocalized with crossover upstream to BCL2 in response to aphidicolin. *BCL2 is shown in green, telomere-specific probe is in red, BrdU shown in cyan and DAPI shown in greyscale*. **C.** Full metaphase spread harboring chromatid breaks at the fragile sites BCL2 and FRA12C1 (white arrows) and a complex rearrangement involving BCL2 within one plate in response to aphidicolin (green arrow). *BCL2 is shown in green, FRA12C1 is in red and DAPI shown in greyscale*. **D.** Examples of complex chromosome fusions with junction points overlapping with crossover events (left) and not overlapping with crossover events (right) from cells exposed to aphidiolin. *BrdU shown in cyan and DAPI shown in greyscale*. **E.** Example of complex rearrangement involved BCL2 region with colocalized chromatid break and crossover and subsequent fusion at the BCL2 region in response to aphidicolin. *BCL2 is shown in green, BrdU shown in cyan and DAPI shown in greyscale*. **E.** The number of cell cycles/S phases a cell experienced during drug treatment can be determined by BrdU-labeling. Cells with no BrdU incorporated correspond to 0 cell cycles, metaphases with equally labeled chromatids correspond to 1 cell cycle, metaphases with differentially labeled chromatids correspond to 2 cell cycles. *BrdU shown in cyan and DAPI shown in greyscale*.

Recently we published a novel method to measure simultaneously successful and repair of replication stress-induced damage at endogenous fragile sites termed SCE-FISH (sister chromatid exchange and fluorescent in situ hybridization) sites [30]. While NHEJ directly rejoins broken DNA ends, HR primes new DNA synthesis from a template DNA, most often the sister chromatid [15, 31]. The intertwined sisters can be resolved in either a non-crossover or crossover orientation, the latter results in the sister chromatids exchanging DNA (Figure 1A-B). With SCE-FISH, the SCE number estimates overall DNA repair by homologous recombination (HR); SCE frequency assesses the rate of repair [32-35]. SCE frequency at specific sites can then be compared using one or more FISH probes (Figure 1B-C). Finally, telomere FISH allows for the assessment of DNA damage and chromosome rearrangements. Currently, only SCE-FISH can assess the capacity of HR-mediated repair and the formation of chromosome rearrangements in the same cell and even on the same chromosome (Figure 1D-E). Importantly, radial fusions and many complex rearrangements also contain two or more centromeres—a situation that can induce defective chromosome segregation and mitotic failure [36].

## Fragile sites and cancer

Fragile sites were originally defined as genomic locations where DNA breaks were visible in condensed mitotic chromosomes. While most fragile sites do not appear to play a direct role in tumor suppression or oncogene overexpression. In mammalian cells, fragile sites were first classified by the method used to induce damage. Rare fragile sites were first identified by breakage in response to folate deprivation or bromodeoxyuridine exposure, while common fragile sites (CFS) were initially discovered by sensitivity to replication inhibitors including aphidicolin, 5-azacytidine, or distamycin A [37-40]. More recently, a class of early replicating fragile sites (ERFS) were discovered in response to acute replication stress with hydroxyurea [4]. Additionally, small molecule inhibitors of the DNA damage checkpoint kinase ATR revealed genomic loci rich in forming non-B DNA structures are prone to DNA breakage [41].

## Sources of fragile site breakage

While damage at rare fragile sites—best characterized by trinucleotide repeat-associated diseases such as Friedrichs ataxia, fragile X syndrome, and Huntington’s disease—can occur in non-dividing cells, the majority of fragile site damage occurs in proliferating cells and requires DNA replication [3, 42-44]. Common fragile sites strongly correlate with late-replicating, origin -poor regions, suggesting replication may not be complete at these loci prior to cell division. However what stalls or delays replication in these regions is still debated, though there are likely multiple causes. CFS are known to form difficult-to-replicate secondary structures [41, 45, 46]. There is also evidence that transcription of very long genes may stall DNA polymerases leading to incomplete replication [47, 48]. However, transcription can also shape nuclear architecture and replication timing [49, 50]. An alternate theory is that transcription affects replication timing at CFSs, rather than creating direct conflicts with moving replication forks [42, 50].

In comparison to CFS, ERFS are origin-rich regions, therefore incomplete replication is unlikely to drive instability observed in mitosis. A host of factors associated with euchromatin and active transcription correlate with ERFS—they overlap with CpG islands, trimethylation of histone at lysine 4, and genes with abundant mRNA production. Many ERFS overlap two or more highly-transcribed genes. A substantial subset of ERFS loci also co-localize with RNA:DNA hybrids—three stranded nucleotide structures that can cause replication stress [4, 51]. Since ERFS replicate early and are origin-rich, we predict that instability stems from impeding replication fork progression rather than incomplete replication.

Though molecularly distinct, ERFS and CFS are sensitive to multiple replication stress-inducing agents. Both experience elevated levels of DNA breaks and rearrangements in response to DNA damage checkpoint inhibition or loss of repair proteins involved in homologous recombination [3, 4, 30, 52-55]. Oncogene overexpression is another potent source of replication stress that induces DNA damage at both ERFSs and CFSs [4, 56]. Finally, both CFS and ERFS are associated with increased mutations, copy number alterations, and rearrangements observed in human cancers [3, 4, 57, 58]. Using SCE-FISH we showed that spontaneous and replication stress-induced SCE is significantly higher at fragile sites than genomic loci with no known predisposition to damage (“cold” sites) [30]. Further, replication stress from checkpoint inhibition or blocking replication progression increased successful and unsuccessful repair at most CFS and ERFS loci examined

## Advantages of SCE-FISH

SCE-FISH assesses successful and unsuccessful repair at endogenous genomic loci, allowing for the direct comparison of the frequency of repaired and unrepaired breaks (Figure 1B, [30]). Further, the rate of SCE formation at independent fragile site loci can be compared to each other and to the total rate of SCE in the same cell sample. This gives two important quantitative comparisons of fragile sites to other loci: it can be assessed as its frequency within a cell population (events per cell), or its frequency to all exchanges (frequency as % of SCEs). With standard 4-color microscopy, three independent FISH probes can be combined in a single hybridization. We found individual cells harboring DNA aberrations at both ERFS and CFS loci, indicating that damage at early and late-replicating regions occurred in the same cell (Figure 1C).

Unlike other techniques, only SCE-FISH also analyzes structural abnormalities allowing for the identification of radial and complex fusions. We found that cells exposed to the replication stress agent aphidicolin often harbored complex rearrangements often contained multiple SCE events (Figure 1D). We hypothesize that structures such as these arise from HR repair failed at the last stage - Holliday junction resolution. Supporting this hypothesis, we found SCE events localizing to fusion junctions in radial and complex rearrangements—a subset of junction sites also overlapped with fragile sites (Figure 1D, E).

Importantly, the BrdU incorporation also measures the extent of DNA replication and cell cycle progression. Metaphase spreads with no BrdU labeling did not traverse S phase during BrdU incubation, cells with equal sister chromatid labeling went through 1 complete S phase, and cells with unequal (but complete) labeling progressed through two full S phases (Figure 1F. This information can help determine two related questions: 1) does DNA damage from replication stress arise by mitotic failure, and 2) what are the temporal requirements of chromosome rearrangement formation?

SCE-FISH can be readily tailored to measure HR at endogenous genomic loci in many contexts, complementing molecular analysis of protein recruitment by ChIP or replication timing and origin usage by OK-Seq or Repli-Seq. Such studies will help define the molecular pathways involved in creating and repairing damage at endogenous fragile sites, as well as oncogenes and loci harboring tumor suppressors. Notably, we found that fragile sites near centromeres (both ERFS and CFS) did not exhibit elevated levels of SCE [30]. These results suggest there may be positional effects governing repair pathway choice, similar to meiosis. Further investigation will reveal if SCE suppression is specific to centromeres or if other chromosomal regions also elicit a similar response. Combined with site-specific DNA breaks created by CRISPR-Cas or TALENs, SCE-FISH could also be harnessed to define the frequency of exchanges in heterochromatin vs. euchromatin, or compare HR frequency between two distinct cell types.

## Limitations

SCE-FISH measures only homologous recombination repair, which results in CO events; NCO products of HR and NHEJ remain unexplored. Further, SCE-FISH measures successful repair in condensed mitotic chromosomes, restricting analysis to 1-5% of the cell population. ~50-100 cells are necessary for accurate quantitation of fragile site repair, which may limit its use in cell cultures or tissues with very low mitotic index. The resolution of FISH is on the megabase-scale. Fragile sites often span regions a megabase or longer, making FISH and ideal method to fully assess damage and repair, however it provides no information concerning repair junctions. We anticipate that in the future ultra-long read sequencing techniques such as PAC-BIO or MinION can be used in tandem to analyze fragile site mutations and rearrangement junctions [59, 60]. SCE-FISH studies directly measures the frequency of repair in a cell population, a component sorely lacking in many next-generation sequencing studies of WT and tumor samples. Thus, functional assays such as SCE-FISH can complement studies characterizing HR-mediated repair, similar to PCR-based assays for the frequency of point mutation.

## Harnessing SCE-FISH in cancer studies

Since fragile site instability can arise from multiple sources, how can we define the underlying cause of replication stress in a tumor? While whole-genome and exome sequencing be helpful, many alterations will have unknown consequences. This is exemplified by variants of unknown significance (VUS) for DNA repair genes like Brca1—little is known about the functional consequence of even relatively common point mutations identified in tumor sequencing [61, 62]. Instead, functional assays such as SCE-FISH can provide more actionable information—fragile site breakage identified by FISH strongly indicates increased replication stress while alterations in SCE frequency can infer if HR is defective [35, 63-67].

Complex chromosome rearrangements are strongly associated with tumorigenesis and are regularly observed in in mature cancers [68-71]. Understanding how cells generate these rearrangements will help us understand cancer etiology, and identify novel ways to exploit their occurrence. In particular they could help structure cancer therapies—in some cases short, acute dosing may be more effective, while other times longer low-level exposure may eliminate tumor cells more completely. Alternatively, chromosome rearrangement analysis would provide insight into potentially “overactive” repair pathways tumor cells rely on for survival.

## Future directions

A current challenge lies in developing assays to identify and quantify DNA repair events resulting in non-crossover HR products and NHEJ. Combining such tools with SCE-FISH will unlock when, where, and how often specific repair pathways are used at the single cell level—and help reveal the complex interplay of genetic and epigenetic factors governing genome stability its role in tumor suppression.
